# Effect of teaching stress management skills on self-esteem and behavioral adjustment in people with somatomotor disabilities

**Published:** 2015

**Authors:** Z Kamalinasab, A Mahdavi, M Ebrahimi, M Vahidi Nekoo, M Aghaei, F Ebrahimi

**Affiliations:** * General Psychology, Islamic Azad University, Science and Research Branch, Tehran, Iran; ** Department of Psychology, Faculty of Psychology and Educational Sciences, University of Payam Noor, Tehran, Iran; *** Clinical Psychology, Islamic Azad University, Science and Research Branch, Tehran, Iran; **** Department of Psychology, Faculty of Psychology and Educational Sciences, University of Tehran, Tehran, Iran

**Keywords:** stress management, self-esteem, behavioral adjustment, somatomotor-physical disability

## Abstract

**Objective:** Psychological interventions for enhancing mental health in those with somatomotor-physical disabilities are vital. The existing research aimed to examine the effect of teaching stress management skills on self-esteem and behavioral adjustment in individuals with somatomotor-physical disabilities.

**Methodology:** The method of the survey was semi-experimental with a pre-test post-test design and a control group. Hence, in Tehran, 40 girls with somatomotor-physical disabilities were selected by using convenience sampling, and they were divided into two groups: control and experiment. Both groups were tested by using a demography questionnaire, Rozenberg’s self-esteem scale, and a behavioral adjustment questionnaire. Afterwards, the test group received lessons on stress management within ten sessions, but the control group received no interventions. Then both groups were post-tested, and the collected data were analyzed by using descriptive and inferential statistics methods through SPSS software.

**Findings:** Findings showed that teaching stress management skills significantly increased self-esteem and behavioral adjustment in girls with somatomotor-physical disabilities (p < 0.001).

**Conclusion:** According to the study, it could be concluded that teaching stress management skills is an effective way to help endangered individuals such as girls who have somatomotor-physical disabilities because it is highly efficient especially when it is performed in groups, it is cheap, and acceptable by different people.

## Introduction

There are a considerable number of disabled people in every society. Disability is an undeniable reality that has existed in all the cultures since past eras. Being non-disabled or disabled is defined based on a biological-mental-social pattern, because disability is the result of a disorder that reduces or inhibits one or more natural bodily roles based on age, gender, and other social or cultural factors [**1**].

In other words, somatomotor-physical disability has been defined as a trauma which limits one or more activities in individuals’ lives (Brown & Turner, 2010); and it can happen in the form of a contemporary or permanent, minor or major, fixed or changeable disability in every society and in every group or class. The operational definition of somatomotor-physical disability is very broad. It includes problems such as difficulty in getting on buses, carrying a five-kilo object, walking on stairs, getting on and off the bed, using a wheelchair, using a cane, using a walker, as well as mental problems such as anxiety, depression, and so forth [**2**]. Hence, it is critical to pay attention to factors that can have a positive effect on the adjustment and positive mental states such as self-esteem that leads to more physical and mental health in those with somatomotor-physical disabilities. 

One of the significant psychological features that help individuals cope with the difficulties efficiently and steadily and keep their achieved skills and values is “self-esteem”. Self-esteem is highly relevant regarding the mental health and personality balance. Self-esteem is a psychological evaluation based on the fact that every individual is valuable and, on the contrary, a person is worthless and unpleasant [**3**]. A low self-esteem bothers the humans’ stability and dynamism, weakens their act, decreases their efficiency, and affects their knowledge and creativity in a negative approach, which is completed by fear. On the contrary, high self-esteem results in positive and constructive strategies, strong motivation, and positive emotional states [**4**]. Therefore, examining the factors that enhance self-esteem in individuals with somatomotor-physical disabilities is of great importance. 

Also, another important factor helping to provide a basis for people, especially the physically challenged ones to put up with the difficulties is “self-esteem”. Achieving social adjustment and a useful, productive relationship with others, and accepting social responsibilities is a major educational goal for every individual especially for those with somatomotor-physical disabilities [**5**]. Achieving social adjustment is the key to social growth, social relationships, high-quality social interactions, social skills, and even mental health [**6**]. Therefore, paying attention to this variable is of great importance to those with somatomotor-physical disabilities, needing studies to examine the ways to enhance adjustment in people with somatomotor-physical disabilities. 

However, many studies have referred to the effect of interventions and teaching psychological lessons on self-esteem and change [**7**]. Hence, it was thought that psychological interventions help individuals with somatomotor-physical disabilities to improve their self-esteem and adjustment. Here, an important factor affecting health, self-esteem change, and welfare is teaching life skills [**8**]. From life skills, learning stress management skills is crucial because physically challenged people need to cope with stressful events effectively. For stress management, individuals are trained to do planning for studies and life affairs, set a logical plan, and allocate the necessary time to unpredictable events [**9**]. Hence, it was thought that teaching stress management skills is needed to improve the mental states in people with somatomotor-physical disabilities, especially their self-esteem and adjustment. Thus, this research was aimed at examining the effect of teaching stress management skills on self-esteem and change in those with somatomotor-physical disabilities in Tehran.

## Methodology

The present study was semi-experimental with a pre-test post-test design and a control group. The statistical community of the present investigation consisted of all the people with somatomotor-physical disabilities in Tehran’s Welfare Organization (autumn, 2016). Because the minimum sample size for experiments was 15 people, for each group, 15 members were selected [**10**]. Afterwards, to increase the statistical competence and manage the probable drops in the number of participants, the sample size was of 20 members (n = 20) for each group. The criteria for entering the present research included a conscious tendency to participate in the study, the ability to take part in sessions and do assignments, being a girl with somatomotor-physical disabilities in a 24-hour rehabilitation center in Tehran, having an age range from 18 to 35, having a minimum education (junior high school). The criteria for exiting the research included the following indexes: 

Not having a tendency to participate in sessions. 

Being absent for more than three meetings.

Not having the ability to take part in sessions and do assignments.

Having severe mental disorders.

Having a medical disorder that affected the result of interventions.

Having a history of psychological and medical therapies that were not part of the present research.

Age in the range of 18 and 35, and not having a minimum education. 

The method of performing the present study included visiting centers covered by the Welfare Organization in Tehran, to which girls with somatomotor-physical disabilities referred to; then the girls were sampled. After ensuring their entrance-exit criteria, 40 girls were randomly selected as an example, each group having 20 members. Afterwards, the girls were given explanations about the study, therapy sessions, and research questionnaires; and when they agreed to take part in the research, they were randomly placed in one of the two groups: experiment and control. Before performing the study, in order to observe the ethical principles of the research and to ensure the presence of girls with somatomotor-physical disabilities, they were asked whether they wanted to participate in the research or not. After that, the experiment group received group lessons on stress management skills within ten sessions, but the control group received no interventions. Finally, both groups were post-tested. The protocol of problem-solving training sessions is presented in **Table 1**. 

The tools used for the present study included a demographic questionnaire, Rozenberg’s self-esteem scale, and a behavioral adjustment questionnaire. 

Demography Questionnaire: Scholars designed this survey to collect personal information from respondents. In this survey, respondents asked questions about items such as age, education, and field of study. 

Rozenberg’s Self-Esteem Scale: Rozenberg’s Self-esteem scale that was made by Rozenberg in 1965, measures the general self-esteem and personal value. This level included ten general items that evaluated life satisfaction and having a good feeling about oneself [**11**]. According to Barnet & Right (2002), Rozenberg’s self-esteem scale (SES) is one of the most common scales for measuring self-esteem, which is a well-recognized measure since, for self-esteem, it uses a concept similar to the concept presented in the psychological theories of oneself. SES was formed to give a general image of positive and negative attitudes to oneself. Using Cronbach’s alpha, Rozenberg (1965) reported the reliability of this survey to be 0.89. This measure has stronger correlation coefficients than Cooper Smith’s self-esteem survey (SEI), and regarding self-esteem, it has greater validity [**12**]. To perform this test, the scale was given to respondents, and they wanted to state their opinion by choosing “I agree” and “I disagree” items. Rozenberg reported the reformation of the scale to be 0.9, and he stated that the scalability of the size was 0.7 [**11**]. For this magnitude, in the first turn, Cronbach’s alpha coefficient was 0.87 and 0.86 for men and women respectively; and in the second turn, it was 0.88 and 0.87 for man and women. The association of the retest was a limit from 0.82 to 0.88, and the internal flexibility factor or Cronbach’s alpha was in a range from 0.77 to 0.88. This scale had an acceptable internal validity (0.77). 

Behavioral Adjustment Questionnaire: This questionnaire was designed and normalized by Sajedi in 1996, and had 78 questions covering three general dimensions: emotion (items 1-7, 13, 16, 21-37, 40, 41, 43, 47-52) and cognition (issues 61-78). Each issue was on a six-point Likert scale (“always” to “never”). The reliability and validity of this questionnaire were shown to be favorable in different studies. Using Cronbach’s alpha, Khodapanahi MK, Khaksar Boldaji MA (2015) [**13**] calculated the reliability of this scale to be 0.91, 0.84, and 0.90 for emotion, cognition, and education, respectively, which were acceptable. Also, Faramarzi S, Asgari K, Taghavi F (2013) [**14**] calculated the validity of the questionnaire, and by using Cronbach’s alpha, the internal correlation of this scale was calculated to be 0.77 for emotion, 0.79 for cognition, and 0.78 for education. Also, in a study, by using Cronbach’s alpha, Khodapanahi MK, Khaksar Boldaji MA (2015) [**13**] calculated the reliability of the questionnaire to be 0.95 for emotion subscale, 0.89 for cognition sub-dimension, and 0.99 for education sub-dimension. A sample of this survey was given in Appendix 2. Additionally, by using Cronbach’s alpha, the validity of the questionnaire was calculated to be 0.91 for variables, which showed a high consistency in the scale. This questionnaire was also examined by some experts to determine validity; its efficacy was calculated to be 0.86 [**14**].

To analyze data, “SPSS-20” software was used. For analyzing the research information in a descriptive statistics level, the statistical method used indexes such as mean, standard variation, frequency, and percentage; and in an inferential statistics level, it used the tests of single-variable and multivariate covariance analysis model. 

**Table 1:** Cognitive-behavioral group therapy training protocol

session	subject
first	Introduction to group members, familiarizing, introduction to stress, stressors, stress responses, and awareness of the effect of stress on the body
second	Awareness of stress effects and understanding this awareness and increasing awareness of physical responses related to stressors
Third	Explanation on the relationship of thoughts, emotions, and bodily senses, and offering numerous examples in different situations
fourth	Introduction and identification of all types of common negative thoughts and cognitive falsifications
fifth	Challenging common negative thoughts and cognitive falsifications and replacing logical thoughts with irrational thoughts
sixth	Training, rehearsal, and performing effective solutions
seventh	Continuing training, rehearsal, and performing effective solutions
eighth	Training, discussion over anger management, decisiveness, time management, recording daily events
ninth	Teaching how to use problem-solving skills in conflicts, discussion over how to say “no”, allocating assignments
tenth	Training and understanding the importance of social support advantages, and a complete review of plans

**Research Findings**

The demographic characteristics of the present sample are presented in **Table 2**.

**Table 2:** Demographic characteristics of respondents

variable	group	frequency	Frequency percentage	Mean and standard deviation
Age	18 to 25 years	15	37.5	27/ 05 ± 6/ 03
	26 to 30 years	12	30	
	30 to 35 years	7	17.5	
	36 to 40 years	6	15	
Level of education	Under diploma	8	20	
	Diploma	9	22.5	
	BA	15	37.5	
	MA	8	20	
Marital status	Single	26	65	
	Married	14	35	

As shown in **Table 1**, the most frequency of individuals participating in the present study was related to people between the ages of 26 and 30, i.e. 12 persons (30 percent). In addition, the least frequency of individuals was related to people between the ages of 36 and 40, i.e. six individuals (15 percent). Also, the average period of the participants in the study was 27.05, and the standard deviation was 6.03. The other data related to the demographic features of the present sample are shown in **Table 2**.

**Table 3:** Descriptive statistics of the scores of research variables in the two groups based on pre-test and post-test

component	index	experiment		control	
		Pre-test	Post-test	Pre-test	Post-test
Self-esteem	Mean	2.61	8.01	3.30	3.45
	Standard deviation	1.04	1.12	0.97	0.99
Emotion dimension	Mean	68.75	94.10	84.20	84.10
	Standard deviation	11.53	12.52	12.09	11.23
Cognition dimension	Mean	50.70	77.25	48.55	48.90
	Standard deviation	5.83	8.42	8.58	8.23
Education dimension	Mean	43.75	76.80	49.95	50.70
	Standard deviation	5.68	11.56	5.32	4.88

As shown in **Table 3**, in the experiment group, the average score of self-esteem, emotion, cognition, and education in the post-test stage increased, compared to the control group. 

**Table 4:** Results of Loin’s test for examining pre-hypothesis of variance consistency, self-esteem, and its behavioral dimensions in the post-test stage

variable	stage	F	Degree of freedom 1	Degree of freedom 2	Significance level
Self-esteem	Posttest	0.077	1	38	0.783
Emotion	Posttest	0.273	1	38	0.604
Cognition	Posttest	0.001	1	38	0.972
Education	Posttest	0.384	1	38	0.539

As presented in **Table 4**, the hypothesis of zero about the equality of variances in both groups in self-esteem and dimensions such as emotion and cognition-emotion was approved. For all the variables of behavioral adjustment, i.e. self-esteem, emotion, cognition-emotion, variances of both groups were equal in the society with no significant difference. Therefore, following Loin’s pre-hypothesis, it was possible to perform a covariance analysis of the results to examine the hypothesis of the study.

**Table 5:** Results of multivariate covariance analysis for post-test scores with pre-test control in self-esteem, emotion, cognition, and education

Test title	value	F	Degree of freedom	Significance level	squared Eta	competence
Pylayy effect	0.864	76.297	3	0.001	0.864	0.95
Wilks Lambda	0.136	76.297	3	0.001	0.864	0.95
Hotelling effect	6.358	76.297	3	0.001	0.864	0.95
Ray’s largest root	6.358	76.297	3	0.001	0.864	0.95

As shown in **Table 5**, a significance level of all tests (p > 0.001) indicated that at least in one of the dependent variables (self-esteem and behavioral adjustment dimensions), there was no significant difference between the two groups. Moreover, based on the eta square, 0.86 percent of the differences observed between individuals were related to the effect of independent variable, i.e. intervention method (teaching stress management skills). On the other hand, because the mathematical competence was 0.95, which was greater than 0.80, the sample size for performing the research was acceptable. The results related to the significant difference of each dependent variable are presented below.

**Table 6:** Results of multivariate covariance analysis for examining the effect of teaching stress management skills on the level of self-esteem and behavioral adjustment dimensions in the post-test stage

Index	Sum of squares	Degree of freedom	Mean of squares	F	Significance level	Squared Eta
Self-esteem	207.025	1	207.025	183.165	0.001	0.828
Emotion	1001.025	1	1001.025	6.658	0.011	0.149
Cognition	8037.225	1	8037.225	115.707	0.001	0.753
Education	6812.101	1	6812.101	86.477	0.001	0.695

Based on the data presented in **Table 6**, since the significance level was p > 0.001, the difference of self-esteem and the dimensions of behavioral adjustment between the two groups were approved. Additionally, 0.82 percent of the changes in the score of self-esteem, 0.14 percent of the variations in the score of emotion, 0.75 percent of the variations in the score of cognition, and 0.69 percent of the differences in the score of education were due to the independent variable (teaching stress management skills). Thus, it can be said that learning stress management skills helps increase self-esteem and behavioral adjustment dimensions in individuals with somatomotor-physical disabilities.

## Discussion and Conclusion

Considering the present research, which was aimed to examine the effect of teaching stress management skills on the self-esteem and adjustment in people with somatomotor-physical disabilities, findings obtained from single-variable and multivariate covariance analysis showed that learning stress management skills had a significant effect on the self-esteem and behavioral change in physically challenged people. It was in congruence with findings obtained from studies conducted by Kazemi N (2015), Soleimani Dinani M, Ali Akbari Dehkordi M, Juybari AA, Moradi A (2012), Safaralizadeh F, Partoazam H, Habibpour Z (2011), Dahaghin V, Atef Vahid MK, Asghar Nejad AA (2015), Mojarrad Kahani AH, Ghanavi S (2013), Haugh CL (2010) [**3**,**5**,**11**,**15**-**17**].

To express their similar findings, Dahaghin, Atef Vahid, and Asghar Nejad (2015) stated that teaching stress management skills makes it possible for physically challenged individuals to nurture necessary abilities to act according to their standards and to achieve goals in a different situation. Also, it expanded personal knowledge and reinforced positive beliefs. All these factors helped prevent mental disorders, leading to an increase in self-esteem [**16**]. However, training helped individuals learn more about themselves, identify their weaknesses and strengths, and try to improve their weaknesses. In conclusion, people accept realities better, come to terms with them in a correct way, which helps them adjust, fight challenges better, reduce mental pressure, and finally increase confidence [**15**]. 

To express his findings, Kazemi N (2015) [**3**] stated “Despite the fact that self-esteem is individuals’ perception of competence, eligibility, and capabilities as well as considerable predictions of performance and satisfaction [**17**]. In some cases, self-esteem might be intensely affected”. For instance, in individuals with somatomotor-physical disabilities, it seems that the central self-assessment is influenced because they have abnormal physical conditions leading to negative assessments. It is mainly because physically challenged people experience losses that are emotionally and physically important. These people face numerous physical, law, economic, mental, and social orientations, obstacles, and prejudices. In other words, disability is not only seen in the people’s bodies, but it is also seen in their thoughts and perceptions in the society [**16**]. Because the conditions of people with somatomotor-physical disabilities increase the mental disorders [**3**], self-esteem is affected. Therefore, noticing the fact that teaching life skills help individuals learn more about themselves, it could increase self-esteem in them. 

Additionally, the idea that teaching stress management skills significantly increases behavioral adjustment dimensions in those with somatomotor-physical disabilities was in congruence with findings obtained from studies conducted by Kazemi N (2015), Soleimani Dinani M, Ali Akbari Dehkordi M, Juybari AA, Moradi A (2012) [**3**,**5**]. To express his similar findings, Kazemi (2015) stated that the educational contents of life skills sessions, which include teaching different dimensions of social issues such as ways to communicate effectively, learning verbal and non-verbal methods of communication, increasing decisiveness, and so forth, could enhance the individuals’ efficiency in social interactions and reduce difficulties. In fact, improvements in social interactions help individuals avoid loneliness, leading to a decrease in the negative attitudes and thoughts. Teaching life skills increase the individuals’ self-efficacy and help them experience a higher social adjustment. Botvin & Griffin (2004) also stated that teaching life skills increases skills related to the social resistance and helps individuals learn how to increase their personal-social eligibility. To express their similar findings, Baghayi Moghadam, Malakpour, Amiri, and Molavi (2012) stated that teaching stress management skills significantly affects the individuals’ adjustability. Because when people gather in a group and feel that others have the same problems with them, they can share their experiences to cope with stress and become more adjustable. On the other hand, teaching life skills helps individuals learn more about themselves and identify their weaknesses and strengths so that they could reinforce their positive points and solve their problems. 

**Acknowledgements**

The authors would like to thank to the officials of the Welfare Organization. Also, the authors would like to thank all the participants in the research.
